# Oral Hyperpigmentation Secondary to Radioactive Iodine Therapy

**DOI:** 10.1210/jcemcr/luaf112

**Published:** 2025-06-09

**Authors:** Rachel M Boice, Christina M Mitchell, Thomas P Sollecito, Roopali Kulkarni

**Affiliations:** Department of Oral Medicine, University of Pennsylvania School of Dental Medicine, Philadelphia, PA 19104, USA; Clinical Medicine in Endocrinology, University of Pennsylvania School of Medicine, Philadelphia, PA 19104, USA; Department of Oral Medicine, University of Pennsylvania School of Dental Medicine, Philadelphia, PA 19104, USA; Department of Oral Medicine, University of Pennsylvania School of Dental Medicine, Philadelphia, PA 19104, USA

**Keywords:** oral hyperpigmentation, radioactive iodine therapy, thyroid carcinoma

## Image Legend

A 75-year-old female individual presented to the oral medicine clinic complaining of dryness and generalized color change in her mouth of 3 months' duration. This began 10 days after radioactive iodine therapy (RAI). She endorsed a complete loss of taste but denied other oral symptoms. She was previously evaluated by her endocrinologist and otorhinolaryngologist with no diagnosis or treatment rendered. Medical history included metastatic papillary thyroid carcinoma, treated by total thyroidectomy, bilateral cervical lymphadenectomy with bilateral modified neck dissection, and RAI (I-131 Rx 97.3 mCi). Whole body scans completed 18 hours and 1 week after RAI revealed radioiodine uptake localization involving the salivary glands and pharynx.

**Figure 1.**
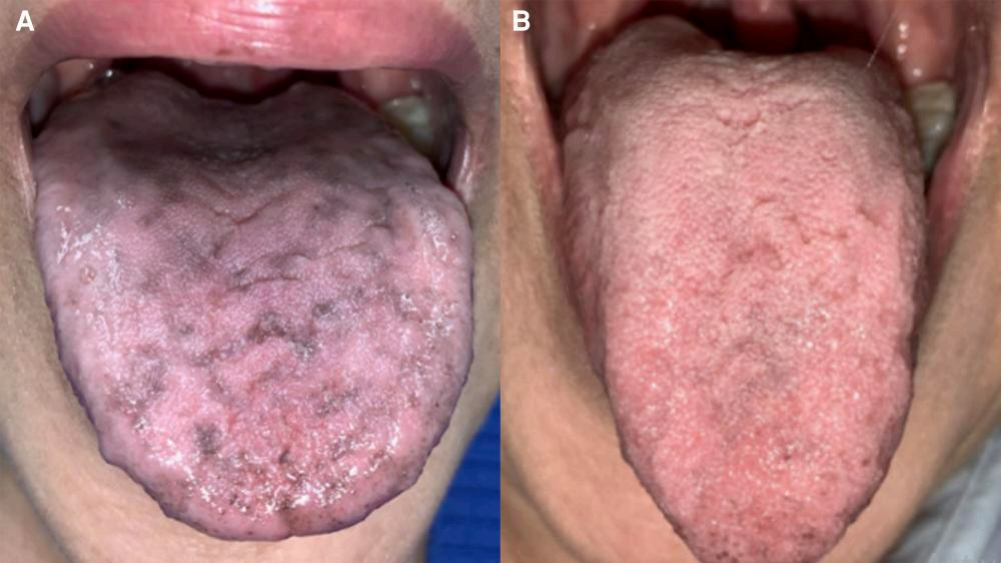
Patient presentation at initial appointment and at three-month follow up.

Intraoral examination was notable for areas of generalized hyperpigmentation bilaterally on the buccal mucosa, labial mucosa, dorsal tongue, and palate (Fig. 1A). Diagnoses were consistent with salivary hypofunction, ageusia, and hyperpigmentation of the oral mucosa in the setting of prior RAI. Alpha lipoic acid 600 mg daily was recommended to the patient for ageusia. Recommendations for salivary hypofunction included salivary gland massage instruction, increased water intake, and use of over-the-counter sialagogues. At the 3-month follow-up visit, the pigmentation had notably decreased (Fig. 1B). This image demonstrates a rare but possible oral adverse effect of RAI therapy [1, 2].

## Data Availability

Data sharing is not applicable to this article as no datasets were generated or analyzed during the current study.
